# Vacuum Plasma Treatment Device for Enhancing Fibroblast Activity on Machined and Rough Titanium Surfaces

**DOI:** 10.3390/dj12030071

**Published:** 2024-03-07

**Authors:** Luigi Canullo, Tullio Genova, Giorgia Chinigò, Roberta Iacono, Paolo Pesce, Maria Menini, Federico Mussano

**Affiliations:** 1 Department of Surgical Sciences (DISC), University of Genoa, Largo R. Benzi 10, 16132 Genoa, Italy; luigi.canullo@unige.it (L.C.); maria.menini@unige.it (M.M.); 2 Department of Life Sciences and Systems Biology, University of Torino, Via Accademia Albertina 13, 10123 Turin, Italy; tullio.genova@unito.it (T.G.); giorgia.chinigo@unito.it (G.C.); 3 Department of Oral and Maxillo-facial Sciences, “Sapienza” University of Rome, Via Caserta 6, 00161 Rome, Italy; iacono.1636694@studenti.uniroma1.it; 4 CIR Dental School, Department of Surgical Sciences, University of Torino, Via Nizza 230, 10126 Torino, Italy; federico.mussano@unito.it

**Keywords:** plasma, titanium, cell adhesion, bioactivation, fibroblast

## Abstract

This study was conducted to compare the effects of an innovative plasma surface treatment device that does not need a gas supply for titanium disks with two different surface topographies: the prototypical machined surface (MAC) and one of the most diffused roughened ones (SL) obtained through grit blasting and acid etching. A total of 200-MAC and 200-SL titanium disks were used. Each group of disks was divided into four sub-groups of 40 samples each that were subjected to five different tests. Among these, 150-MAC and 150-SL were considered the test group, and they were treated with plasma for 15, 30, and 60 s after being removed from the sterile packaging. On the other hand, 50-MAC and 50-SL were considered the control group, and they were only removed from sterile plastic vials. The samples were analyzed to evaluate the capability of the plasma treatment in influencing protein adsorption, cell adhesion, proliferation, and microbial growth on the test group disks when compared to the untreated disks. Protein adsorption was significantly enhanced after 20 min of plasma treatment for 15 and 30 s on the MAC and SL disks. Plasma treatment for 15 and 30 s significantly increased the level of adhesion in both treated samples after 30 min. Furthermore, the MAC samples showed a significant increase in cell adhesion 4 h after plasma treatment for 15 s. The SEM analysis highlighted that, on the treated samples (especially on the MAC disks), the cells with a polygonal and flat shape prevailed, while the fusiform- and globular-shaped cells were rare. The encouraging results obtained further confirm the effectiveness of plasma treatments on cell adhesion and fibroblast activity.

## 1. Introduction

After implant placement, whatever the planned loading protocol, the insertion of the abutment is an important phase of the rehabilitation. It becomes a real surgical act when the abutment is inserted on a submerged implant fixture that is already osseointegrated [1]. It necessarily produces peri-implant stress and trauma, and, in these circumstances, there is temporary exposure of the implant to the oral environment. Furthermore, as demonstrated by Hermann et al., bone remodeling may occur in the hard tissues around the implant [2].

Some strategies were tested in terms of enhancing abutment integration, as well as in limiting peri-implant soft tissue disruption and marginal bone resorption. Several authors have suggested that, by reducing the number of interventions such as abutment detachments and reattachments, peri-implant resorption can be minimized. In fact, one-time definitive abutment placement has shown a biological and clinical advantage by preserving peri-implant tissues [3,4,5].

Abutments have a direct interaction with the tissues surrounding an implant, especially with connective tissue, and they might also affect the marginal bone level. In fact, titanium surface characteristics significantly affect the behavior of cells in peri-implant tissues [6]. In particular, the titanium wear microparticles that are derived from industrial manufacturing processes and atmospheric pollutants—as well as the contaminants derived from the packaging and stockage processes or debris and bacteria deriving from customization processes in the dental lab—can contaminate the abutment surface and consequently hinder the cell response [7].

In addition, as has been demonstrated by various authors, the implant–abutment junction might become the site of colonization and encourage the growth of bacteria, thus triggering mucositis [8,9]. Moreover, titanium wear microparticles could play a role in the stimulation of potent innate and adaptive immune responses, and they have also been found to be related to the activation of osteoclastogenesis [10]. Therefore, before abutment placement, cleaning and decontamination processes must be performed. The microbiome plays an important role in cell/abutment interaction, mostly at an early stage, since the microbiological environment may have to compete for the space in connective tissues, thus hindering the abutment integration process. When the abutment is successfully integrated, the mucosal attachment acts as a barrier to the bacteria that are responsible for bone resorption [11].

Clean abutment surfaces may guide better cell adhesion and tissue connective attachment while preventing the inflammation of peri-implant tissues and their possible resorption, which can be achieved by using different procedures like ultrasonic baths, steam cleaning, ultraviolet light photo-functionalization, or the plasma of argon [8,12,13,14,15].

Plasma treatment, which has relied on the research studies available in the literature for more than a decade, not only produces effective decontamination of the abutments, but it also offers additional biological benefits that result in clinically increased connective tissue adhesion to the abutment and lower MBL [13,16].

Plasma cleaning induces the bioactivation of titanium surface abutments. The bioactivation through bio-physical methods increases abutment wettability through the augmentation of surface free energy, which inversely correlates with the presence of contaminants on the abutment surface. In a clinical scenario, the increased surface wettability induces a stronger fibroblast adhesion, even at the initial stages of the healing process. This is demonstrated by the presence of pseudopodia and a better tissue connective organization with higher collagen fiber density and a higher number of oblique fibers, as shown by Garcia et al. [17].

The evident effectiveness of plasma treatment has urged clinicians, researchers, and companies to optimize abutment cleaning protocols, thereby taking advantage of the innovations of the new devices. This in vitro study was conducted with the aim of testing the treatment of implant abutments with a plasma surface treatment device. The null hypothesis tested was that no differences are present between the titanium surfaces treated with plasma and those that are not treated regarding microbial growth, protein adsorption, and cell adhesion, proliferation, and morphology. The differences between machined and moderately rough surfaces were also evaluated.

## 2. Materials and Methods

### 2.1. Study Design and Sample Description

**Figure 1:**
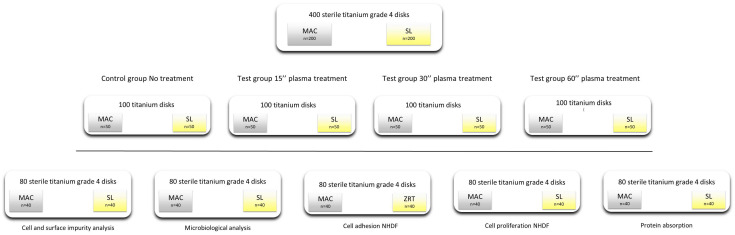
Flow diagram of the study experiments.

To perform the tests, four hundred serially numbered, sterile c.p. (Grade 4) Ti disks with a 6 mm diameter were prepared (Alpha-Bio Tec, Tel Aviv, Israel). They were treated in order to obtain two different surface topographies (200 machined (MAC) and 200 moderately rough (SL)). Every group of titanium disks was divided into five sub-groups of 40 samples, and each of them underwent five different tests (Figure 1).

N was set at 10 in accordance with the previously published article on this topic [13].

To emulate clinical environmental conditions, all of the procedures were conducted within a surgically sterile field at room temperature within a clinical setting. Test samples were extracted from their plastic vials and promptly inserted using an insertion tool into the plasma surface treatment device (ACTILINK, Plasmapp, Seoul, Republic of Korea), which is designed to generate a vacuum from 5 to 10 Torr, as well as to discharge plasma on the surface of a test sample [18,19,20]. Control samples were solely extracted from the plastic sterile vials. To replicate a surgical workflow, samples were left 15 s in a clinical environment, and then the treated samples were treated for 15, 30, or 60 s of plasma treatment. Next, the temperature of the samples was measured (Table 1).

#### 2.1.1. Microbiological Analysis

After the experimental period was concluded, the samples were transferred into a Biosafety Level 2 (BSL2) hood. Subsequently, the implants were placed into Snap-Cap tubes (Sarstedt AG & Co, Nümbrecht, Germany) that contained 5 mL of LB Broth Gibco^®^ (Thermo Fisher, Waltham, MA, USA). Optical density at 600 nm served as the measurement for bacterial growth. All the specimens under consideration were then incubated in 5 mL of LB Broth Gibco^®^ (Thermo Fisher, Waltham, MA, USA) at 37 °C for 24 h with constant orbital shaking at 200 rpm. The optical density at 600 nm (OD 600) was assessed using a Jenway 6300 spectrophotometer (Keison, Chelmsford, UK).

For the determination of the colony-forming units per milliliter (CFU/mL), 1 mL of incubated LB Broth was plated onto LB Agar Petri dishes and then incubated at 37 °C. After 24 h, the colonies were enumerated.

#### 2.1.2. Protein Adsorption

The titanium disks were (a) incubated after treatment for either 20 min or 4 h in a 2% fetal bovine serum (FBS) solution in a phosphate-buffered saline (PBS) at 37 °C for 30 min; (b) rinsed twice in PBS; (c) eluted in Tris Triton buffer (10 mM Tris (pH 7.4), 100 mM of NaCl, 1 mM of EDTA, 1 mM of EGTA, 1% Triton X-100, 10% Glycerol, and 0.1% SDS) for 10 min. The total protein content was quantified utilizing the Pierce™ BCA Protein Assay Kit (Life Technologies, Carlsbad, CA, USA) in accordance with the manufacturer’s guidelines.

#### 2.1.3. Cell Model

For this investigation, primary normal human dermal fibroblasts (NHDFs) (Pro-moCell, Heidelberg, Germany) were used. Cells were cultured at 37 °C in 5% CO_2_ with a saturating humidity in Dulbecco’s modified Eagle’s medium (DMEM) (Thermo Fisher), which was supplemented with 10% fetal bovine serum (FBS) (Thermo Fisher), 2 mM of GlutaMax (Gibco), and 50 µg/mL of Gentamicin (Thermo).

#### 2.1.4. Cell Adhesion

To evaluate cell adhesion, the titanium samples were placed in 48-well plates (BD, Milan, Italy). On each pre-treated disk, 3 × 10^3^ cells were seeded in 1 mL of the growth medium after a trypsin treatment for 3 min. The 48-well plates were kept at 37 °C and 0.5% CO_2_ for either 20 min or 4 h. The cells were rinsed twice with PBS, both before and after fixation in 4% paraformaldehyde in PBS for 15 min at room temperature. Subsequently, they were stained with 1 μM of DAPI (Molecular Probes, Eugene, CA, USA) for 15 min at 37 °C to visualize the cell nuclei. The samples were examined using a Nikon Eclipse T-E microscope with a 4X objective. The counting of cell nuclei was conducted using ImageJ (NIH) software (version 1.54h) utilizing the “Analyze particles” tool.

#### 2.1.5. Cell Proliferation

The cells were seeded at a density of 2500 cells per well in 24-well culture dishes, and their viability was evaluated after 24 h using Cell Titer GLO (Promega, Milan, Italy) in accordance with the manufacturer’s instructions.

#### 2.1.6. Cell Morphology and Focal Adhesion

The cells were seeded onto titanium disks. The concentration was of 104 cells per well in a 48-well plate (BD, Milan, Italy), and these were then maintained under growth conditions. After 1, 4, and 24 h, the titanium samples were rinsed with PBS before the fixation of the cells with 4% paraformaldehyde in PBS for 15 min. Following PBS washing, the cells were permeabilized using 0.1% Triton X-100 (Sigma-Aldrich, St. Louis, MO, USA) in PBS. Subsequently, the cells were stained with rhodamine-phalloidin (Life Technologies) to visualize the cytoskeleton and 1 μM of DAPI (Molecular Probes, Eugene, CA, USA) to label the nuclei. Focal adhesions were identified using an anti-Paxillin N-Term 04-581 antibody from Millipore (Merk, Darmstadt, Germany). Images were captured using a Nikon Eclipse Ti-E microscope equipped with the following various objectives: Nikon Plan 10X/0.10, Nikon Plan Fluor 40X/0.75, and Nikon Plan Apo VC 60X/1.40 (Nikon Instruments, Amsterdam, The Netherlands).

#### 2.1.7. Cell Morphology at SEM

The cells were grown on disks, as described above, and fixed with 4% paraformaldehyde and 2% glutaraldehyde in PBS. After thoroughly washing them in the same buffer, the samples were post-fixed in 1% osmium tetroxide for 45 min in the dark, and they were then rinsed in PBS. The samples were rapidly washed in distilled water and then gradually dehydrated in ethanol to 100%. The disks were air-dried under a fume hood, sputter-coated with a thin layer of gold and observed under a Gemini 300 field emission SEM (Carl Zeiss AG, Jena, Germany).

### 2.2. Statistical Analysis

The data were evaluated using GraphPad Prism 10.0.0 (GraphPad Software, Inc., La Jolla, CA, USA). Each test was replicated at least three times. Statistical analysis was conducted employing either the non-parametric Mann–Whitney test or the Student’s *t*-test. A *p*-value below 0.05 was deemed statistically significant.

## 3. Results

### 3.1. Temperature

In Table 1, it is possible to appreciate the temperature recordings after the different plasma treatment timings of the titanium samples and the time required to reach 37 °C were conducted.

### 3.2. Microbiological Analysis (Table 2)

Data on the microbiological analysis are reported in Table 2.

**Table 2:** Data relative to the microbiology assay performed on the titanium samples treated with plasma for 15, 30, and 60 s.

Microbiology Assay			
	Mean	Std. Dev.	*p* Value vs. CTRL
CTRL contaminated	697.25	415.913	0.009
CTRL	5	5.09902	
15″	7.75	8.341663	0.94
30″	7.25	7.932003	0.8017
60″	7.75	9.287088	0.7824

**Figure 2:**
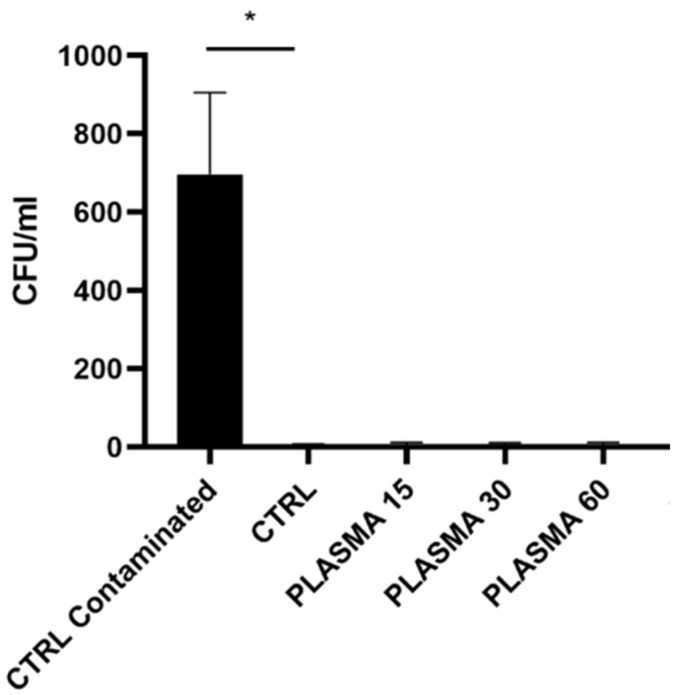
The assessment of the sample contaminations involved quantifying the number of colony-forming units per milliliter (CFU/mL) of the LB Broth medium, a medium that was incubated with the tested disks following exposure to controlled environmental conditions for post-plasma treatment durations ranging from 15 to 100 s. * indicates statistical significance vs. the CTRL surface (where a *p* value of < 0.05 is considered statistically significant).

As shown in Figure 2, exposing the samples to controlled environmental conditions (due to the plasma and subsequent time required to reach 37 °C) for 15 to 100 s did not alter their sterility. The contaminated control specimen was found to be significantly higher contaminated than the ctrl one. 

### 3.3. Protein Adsorption (Table 3)

Data on the protein adsorption are reported in Table 3.

**Table 3:** Data relative to the protein adsorption for the MAC and SL titanium samples 20 min and 4 h after plasma treatment for 15, 30, and 60 s.

Protein Adsorption MAC 20 min				Protein Adsorption SL 20 min			
	Mean	Std. Dev.	*p* Value vs. CTRL		Mean	Std. Dev.	*p* Value vs. CTRL
CTRL	0.06325	0.01584		CTRL	0.15325	0.022853	
15″	0.1438	0.008614	0.0007	15″	0.198	0.039302	0.0282
30″	0.1454	0.0123	0.0008	30″	0.19725	0.008655	0.0326
60″	0.102	0.019374	0.285	60″	0.1605	0.01578	0.7756
Protein adsorption MAC 4 h				Protein adsorption SL 4 h			
	Mean	Std. Dev.	*p* value vs. CTRL		Mean	Std. Dev.	*p* value vs. CTRL
CTRL	0.238	0.027731		CTRL	0.269667	0.029501	
15″	0.249333	0.03722	0.855	15″	0.267	0.023643	0.9636
30″	0.242667	0.030534	0.891	30″	0.272333	0.010263	0.855
60″	0.229667	0.011504	0.8193	60″	0.278667	0.020793	0.5526

To evaluate the capability of a plasma treatment in affecting the protein adsorption on titanium surfaces, different titanium topographies (MAC and SL) were treated with plasma for 15, 30, and 60 s.

**Figure 3:**
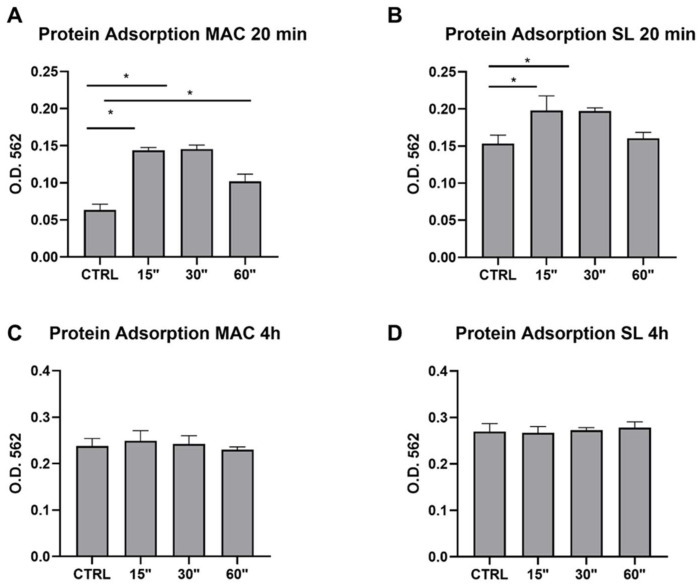
The protein adsorption was evaluated on MAC and SL samples at 20 min and 4 h, as well as on the plasma treatments for 15, 30, and 60 s. The level of protein adsorption was evaluated using a BAC assay. Values represent the mean ± SEM. * indicates statistical significance vs. the CTRL surface (where a *p* value of < 0.05 is considered statistically significant).

As it appeared to be the case from the quantitative assays performed, a plasma treatment for 15 and 30 s significantly improved the protein adsorption in both the MAC and SL titanium topographies after 20 min. However, this difference was no longer appreciable at 4 h (Figure 3).

### 3.4. Cell Adhesion (Table 4)

Data on cell adhesion are reported in Table 4.

**Table 4:** Data relative to the cell adhesion assays performed on the MAC and SL titanium samples at 30 min and 4 h after plasma treatments for 15, 30, and 60 s.

Adhesion MAC 30 min				Adhesion SL 30 min			
	Mean	Std. Dev.	*p* Value vs. CTRL		Mean	Std. Dev.	*p* Value vs. CTRL
CTRL	109.6	16.16478		CTRL	152.5	39.128	
15″	170	12.24745	0.0121	15″	218.75	24.12986	0.0109
30″	173.75	18.83923	0.0121	30″	211.25	35.84573	0.0335
60″	97.5	9.949874	0.6654	60″	151	44.29447	0.7647
Adhesion MAC 4 h				Adhesion SL 4 h			
	Mean	Std. Dev.	*p* value vs. CTRL		Mean	Std. Dev.	*p* value vs. CTRL
CTRL	233.5	24.90649		CTRL	232.25	23.27194	
15″	284.75	15.6285	0.0392	15″	263.75	26.28529	0.1987
30″	261	33.33667	0.2437	30″	255.5	52.79836	0.4196
60″	200	15.40563	0.3698	60″	247	40.47221	0.4918

**Figure 4:**
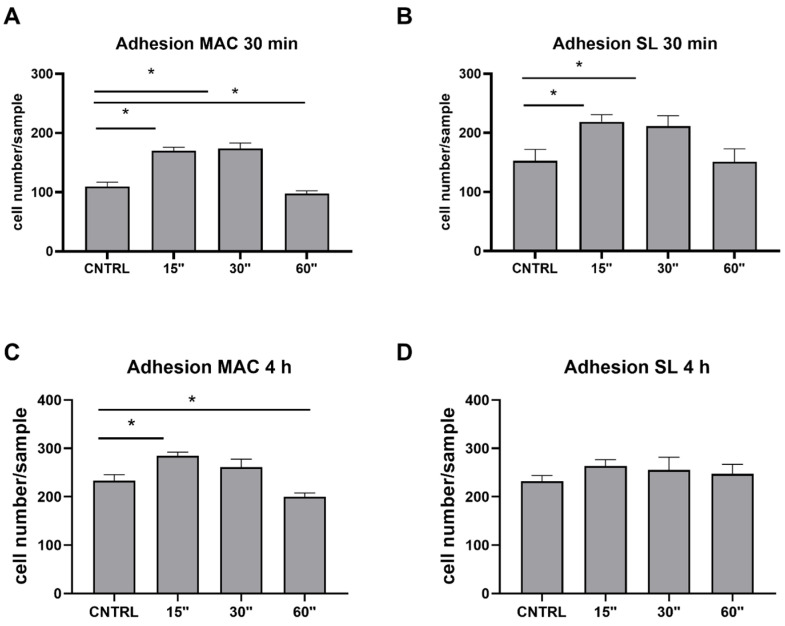
The cell adhesion was assessed on the MAC and SL samples at 30 min, as well as on the 4 h post-plasma treatment durations of 15, 30, and 60 s. The extent of the cell adhesion was quantified by enumerating the number of adherent cells per field. The reported values represent the mean ± standard error of the mean (SEM). Statistical significance compared to the control (CTRL) surface is denoted by * for *p* values less than 0.05.

To test if and how plasma may affect cell responses, a cell adhesion assay was performed on the Ti samples subjected to plasma treatment. As shown in Figure 4, when considering a short time of adhesion (30 min Figure 4A,B), plasma treatments for 15 and 30 s significantly increased the level of adhesion in both the MAC and SL samples. When focusing on a longer time, such as 4 h of adhesion (Figure 4C,D), it was possible to appreciate a significant increase in adhesion in the MAC sample when only using a 15 s plasma treatment.

### 3.5. Viability (Table 5)

Data on the cell proliferation are reported in Table 5.

**Table 5:** Data relative to the cell proliferation assays performed on the MAC and SL titanium samples 24 h after plasma treatments for 15, 30, and 60 s.

Proliferation MAC 24 h				Proliferation SL 24 h			
	Mean	Std. Dev.	*p* Value vs. CTRL		Mean	Std. Dev.	*p* Value vs. CTRL
CTRL	400.25	16.74067		CTRL	431.25	59.71809	
15″	403	37.63863	0.9761	15″	458	81.25269	0.6326
30″	370	58.0804	0.4024	30″	449.5	72.8217	0.8111
60″	357.5	40.47633	0.1064	60″	410	63.35087	0.5501
UV	386.5	27.87472	0.5699	UV	413.25	35.10342	0.6757

**Figure 5:**
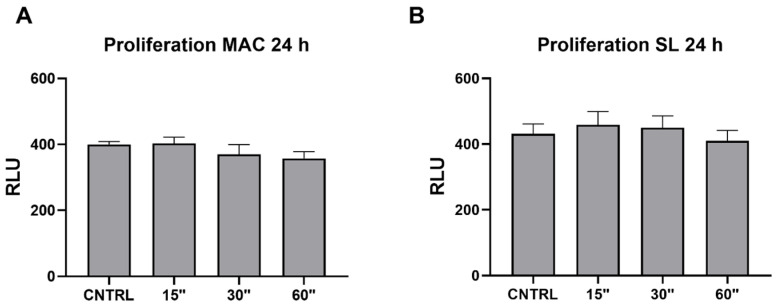
The cell proliferation was assessed on the MAC and SL samples at 24 h post the plasma treatment durations of 15, 30, and 60 s. Evaluation of the proliferation levels was conducted using a luminometric cell titer glo assay. Values are presented as the mean ± SEM.

To evaluate the biological effect of plasma on the cell proliferation, viability assays were performed. Interestingly, no significant difference was observed in any of the considered conditions or samples (Figure 5).

### 3.6. Cell Morphology

**Figure 6:**
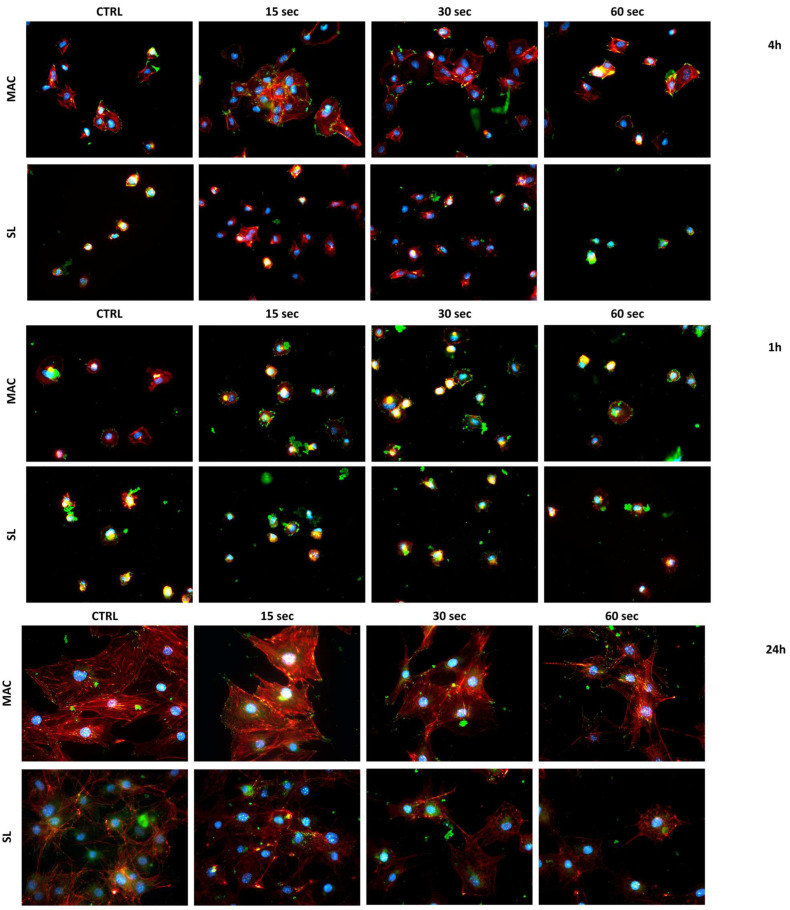
Representative images depicting the morphology of the cells. The fluorescence photomicrographs captured the cells seeded on the MAC and SL samples at 1, 4, and 24 h post the plasma treatment durations of 15, 30, and 60 s. The cells were stained to visualize the nucleus (DAPI, blue), actin filaments (rhodamine-phalloidin, red), and focal adhesions (paxillin, green).

As can be appreciated qualitatively in Figure 6, the cells showed a greater distribution of focal adhesion points on the plasma-treated samples (paxillin) in green after 1 h from seeding. This was found to especially be the case in the 15 and 30 s plasma treatments, and it was most visible in the MAC samples. When considering a longer adhesion time (4 h), a greater spread area was also evident in the samples treated for 15 and 30 s with plasma. Also in this case, this phenomenon was more evident in the MAC samples.

When considering a much longer adhesion time (24 h), the differences in the morphology and focal adhesion points found between the different treatments and the control were no longer evident.

### 3.7. Cell Morphology at SEM

The SEM analysis showed that the vast majority of cells grown on plasma-treated MAC samples were flattened and polygonal in shape. Spindle-shaped cells were observed infrequently, while globular-shaped ones were found only sporadically and were almost completely absent. In most cases, the cells were well spread and featured extended lamellipodia and thin filopodia that were spreading in all directions, thus suggesting satisfactory growth and good adhesion to the disk.

**Figure 7:**
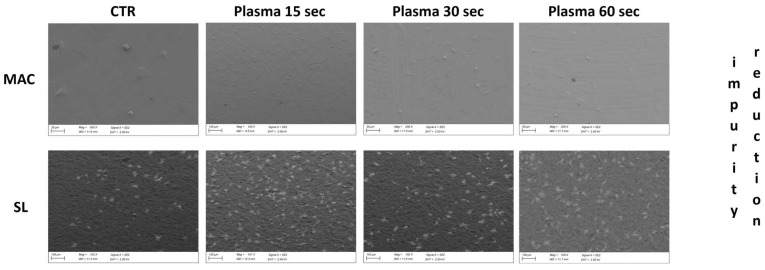
Representative pictures of the post-op conditions of the discs with cell growths at different time points. Quantitatively significant differences can be detected between the control and bio-active surfaces.

**Figure 8:**
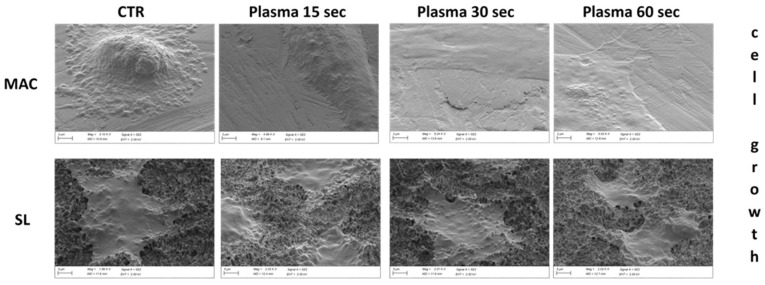
Representative pictures of the cell growth at different time points. Globous cells can be detected on the control machined surface, while spread arrangement can be seen on the bioactivated surfaces at different time points.

In the control samples, most of the cells were also polygonal in shape, but fusiform- and globular-shaped cells were observed more frequently than in the treated samples. In addition, many of the polygonal-shaped cells had a more spherical appearance than those commonly found in the plasma-treated discs. In fact, the polygonal cells grown on the control disc often displayed a markedly swollen and raised area near the central nuclear region, while the more peripheral regions were well flattened, thus suggesting a delay in spreading when compared to the treated samples (Figure 7 and Figure 8).

## 4. Discussion

The decontamination of an abutment surface is an important process through which to remove metallic and bacterial contaminants, thus improving the abutment integration, limiting the onset of inflammation in the peri-implant tissues, and ensuring marginal bone resorption.

Plasma treatment, as already demonstrated in previous studies, allows one to achieve all the aforementioned objectives through the activation of the metallic electronic mantel while also preserving the integrity of the titanium surface effectively [14].

This in vitro study was aimed at testing a novel plasma treatment device that operates with atmospheric gases that are mostly composed of nitrogen and oxygen, and it also does not require any additional gas such as argon, which applies for the devices so far tested in the literature. This new plasma surface treatment device generates a vacuum by removing 99% of atmospheric gases to obtain an optimized condition for discharging plasma, and it has been used previously for implant fixtures to attain enhanced osteoblast activity, as well as improved osseointegration performance [21].

Promising results were observed after the plasma treatment of the SLA and MAC disks with respect to the negative control. The examined plasma device ensured a uniform treatment of the disks and modified their surface topography, thus influencing the biological response through bioactivation.

Here, we demonstrated that the time required for reaching 37 °C after plasma treatment in controlled environmental conditions did not compromise the sterility of the implant compared to the control conditions. Furthermore, as ascertained from the SEM images, the elevations in temperature appeared to elicit no discernible surface damage or alteration to the abutments. This observation aligns with the findings from prior studies on similar air-based plasma devices [19]. It is widely known that microscopic and nano-scale modifications of titanium surfaces can affect the behavior and remodeling of hard and soft tissues, even in the early stages of wound healing [22].

As already demonstrated, plasma bioactivation is able to increase the surface energy of the abutment, hence reducing the contact angle of polar solvents, as well as enhancing the wettability of the surface and making it more hydrophilic. Surface wettability, by improving the interaction with the blood clot, increases the adsorption of proteins, such as fibronectin and vitronectin, with a positive effect on cellular adhesion, thus leading to spreading and proliferation [14,18,19,20].

In accordance with the literature, this study showed that the use of plasma for 15 and 30 s improved the protein adsorption in both the MAC and SL samples after 20 min when compared to the negative control. For this kind of experiment, we did not use isolated proteins (as fibronectin), preferring instead to use a FBS solution to better simulate the complexity of proteins in a physiological environment [23].

This study also showed that fibroblast adhesion is significantly increased after plasma treatment for 15 and 30 s in both samples in the early stages of wound healing (20 min after treatment), but it also showed that the statistical difference with the negative control group tended to flatten out after 24 h. The bio-efficacy of the plasma, though it was appreciable in both the trend of protein adsorption and cell adhesion, disappeared after 24 h due to the saturation effect because of the diameter of the titanium disk. The present results are consistent with other studies, and they suggest that plasma bioactivation induces a stronger fibroblast adhesion on abutment surfaces (even in the initial stages of the treatment [13,14]). The clinical advantage of bioactivation is not only a quantitative one related to a greater number of adherent cells, but also a qualitative one. The latter is due to the morphology of the adherent cells, as it is perceivable from the SEM images. Meanwhile, a flat arrangement was observed in the control group, and a spread arrangement was shown in the bioactivated samples.

A possible explanation for this is that the cells are driven by three-dimensional geometry toward faster differentiation, which results in better cell/abutment integration. This conclusion would be consistent with the paper by Canullo et al. 2021 (even in the initial phase [24]). The main limitation of the “quantitative” endpoint was found to be related to the impossibility of the cells adopted in the study for stratification. However, clinical relevance could be suggested by the “qualitative” endpoints that highlighted the faster cells spreading on the treated disks when compared to the untreated disks. Next to the treated abutments, a wider band of connective tissue formed faster and with stronger contact than the controls, and it also played a crucial role in forming a protective seal around the underlying peri-implant bone, thus minimizing MBL.

Although the quantitative plateau was reached after 24 h, the qualitative advantage remained remarkable. Indeed, the cell spreading was significantly evident and might have produced a better layering than the untreated surfaces. The advantages became evident within 24 h. This was a critical timing because the bacteria strongly interfered with the soft tissue adhesion during this time period [25].

This study further confirmed the efficacy of plasma treatments. The main advantage of the plasma device studied here was the reduced action time required. In previously analyzed devices, the active effect of plasma was generated after 12 min, and this was achieved using the additional processing gas of argon. It is important to emphasize that the tested plasma device provides its maximum effect with a plasma treatment of 15–30 s. Based on previous studies, plasma treatments can chemically modify metal surfaces [26]. Certain analyses, such as EDS and AFM, have demonstrated the effectiveness of plasma treatment in eliminating organic contaminants and oxidizing the metal surface [26]. Therefore, we assumed that there is an optimal range of these cleaning and oxidation processes that enhance protein adsorption and cell adhesion. As indicated by our results, exceeding this optimal threshold may result in counterproductive effects, thus underscoring the significance in identifying the ideal treatment conditions required to attain the desired outcomes. Moreover, for the first time, a vacuum plasma treatment device not fueled by argon gas was used. Therefore, this device represents a clear practical advantage not only in terms of “clinical” time, but also in terms of safety regulation since a strict regulatory burden disciplines the storage of compressed/liquefied gases in workplaces.

## 5. Conclusions

The intrinsic nature of an in vitro study is a limitation and clinical trials are needed to confirm these results in an oral environment. However, based on the present outcomes, the plasma device herein applied—given its effectiveness, as well as its ease and safety of use—can be considered a valuable device to be used for daily abutment decontamination processes.

## Figures and Tables

**Table 1:** Temperature measurements of the plasma-treated titanium samples (MAC and SL) for 15, 30, and 60 s, as well as the relative time required to reach 37 °C.

	MAC				SL			
	CTRL	15″	30″	60″	CTRL	15″	30″	60″
Temperature in °C	25	39	47	51	25	39	46	50
Seconds to 37 °C		60	90	102		60	88	99

## Data Availability

The raw data supporting the conclusions of this article will be made available by the authors on request.
